# Improved Inhibitors Targeting the Thymidylate Kinase of Multidrug-Resistant *Mycobacterium tuberculosis* with Favorable Pharmacokinetics

**DOI:** 10.3390/life15020173

**Published:** 2025-01-25

**Authors:** Souleymane Konate, Koffi N’Guessan Placide Gabin Allangba, Issouf Fofana, Raymond Kre N’Guessan, Eugene Megnassan, Stanislav Miertus, Vladimir Frecer

**Affiliations:** 1 Laboratoire de Physique Fondamentale et Appliquée (LPFA), University of Abobo Adjamé (Now Nangui Abrogoua), Abidjan 02, Côte d’Ivoire; skonate2@yahoo.fr (S.K.); megnase@gmail.com (E.M.); 2 Physics Pedagogical Unit, Laboratory of Environmental Science and Technology, University Jean Lorougnon Guédé, Daloa Bp 150, Côte d’Ivoire; 3 Department of Medical Physics, University of Trieste and International Centre for Theoretical Physics (ICTP), 34151 Trieste, Italy; 4 Laboratoire de Cristallographie-Physique Moléculaire, Université Félix Houphouët-Boigny, Abidjan 22, Côte d’Ivoire; 5 Laboratoire de Chimie Organique Structurale et Théorique, Université Félix Houphouët-Boigny, Abidjan 22, Côte d’Ivoire; 6 International Centre for Applied Research and Sustainable Technology, 84104 Bratislava, Slovakia; 7 International Centre for Theoretical Physics, Strada Costiera 11, 34151 Trieste, Italy; 8 Department of Biotechnologies, Faculty of Natural Sciences, University of Ss. Cyril and Methodius, 91701 Trnava, Slovakia; 9 Department of Physical Chemistry of Drugs, Faculty of Pharmacy, Comenius University Bratislava, 83232 Bratislava, Slovakia

**Keywords:** tuberculosis, *Mycobacterium tuberculosis*, thymidylate kinase, molecular modelling, MM-PBSA, QSAR model of inhibition in vitro, 3D-QSAR pharmacophore, virtual combinatorial library, in silico screening, molecular dynamics, prediction of pharmacokinetic profiles

## Abstract

This study aims to design improved inhibitors targeting the thymidylate kinase (TMK) of *Mycobacterium tuberculosis* (*Mtb*), the causative agent of infectious disease tuberculosis that is associated with high morbidity and mortality in developing countries. TMK is an essential enzyme for the synthesis of bacterial DNA. We have performed computer-aided molecular design of *Mtb*TMK inhibitors by modification of the reference crystal structures of the lead micromolar inhibitor TKI1 *1-(1-((4-(3-Chlorophenoxy)quinolin-2-yl)methyl)piperidin-4-yl)-5-methylpyrimidine-2,4(1H,3H)-dione* bound to TMK of *Mtb* strain H37Rv (PDB entries: 5NRN and 5NR7) using the computational approach MM-PBSA. A QSAR model was prepared for a training set of 31 *Mtb*TMK inhibitors with published inhibitory potencies (${IC}_{50}^{exp}$) and showed a significant correlation between the calculated relative Gibbs free energies of the *Mtb*TMK–TKIx complex formation and the observed potencies. This model was able to explain approximately 95% of the variation in the in vitro inhibition data and validated our molecular model of *Mtb*TMK inhibition for the subsequent design of new TKI analogs. Furthermore, we have confirmed the predictive capacity of this complexation QSAR model by generating a 3D QSAR PH4 pharmacophore-based model. A satisfactory correlation was also obtained for the validation PH4 model of *Mtb*TMK inhibition (R^2^ = 0.84). We have extended the hydrophobic *m*-chloro-phenoxyquinolin-2-yl group of TKI1 that can occupy the entry into the thymidine binding cleft of *Mtb*TMK by alternative larger hydrophobic groups. Analysis of residue interactions at the enzyme binding site made it possible to select suitable building blocks to be used in the preparation of a virtual combinatorial library of 28,900 analogs of TKI1. Structural information derived from the complexation model and the PH4 pharmacophore guided the in silico screening of the library of analogs and led to the identification of new potential *Mtb*TMK inhibitors that were predicted to be effective in the low nanomolar concentration range. The QSAR complexation model predicted an inhibitory concentration ${IC}_{50}^{pre}$ of 9.5 nM for the best new virtual inhibitor candidate TKI 13_1, which represents a significant improvement in estimated inhibitory potency compared to TKI1. Finally, the stability of the *Mtb*TMK–inhibitor complexes and the flexibility of the active conformation of the inhibitors were assessed by molecular dynamics for five top-ranking analogs. This computational study resulted in the discovery of new *Mtb*TMK inhibitors with predicted enhanced inhibitory potencies, which also showed favorable predicted pharmacokinetic profiles.

## 1. Introduction

Tuberculosis is a highly contagious airborne disease and one of the top causes of death worldwide [1]. In 2024, the World Health Organization estimated that 10.8 million people developed tuberculosis worldwide, including 2.55 million in the African region countries [2]. The disease affects people of all ages and is prevalent in many developing countries. The tuberculosis incidence rate of 134 new cases per 100,000 population per year in 2024 has increased from 129 cases in the year 2020 [2]. In the Ivory Coast, the incidence of tuberculosis is 103 cases per 100,000 inhabitants, including all forms of the disease. In 2015, a total of 23,000 cases of tuberculosis were detected, of which 24% were formed by co-infections of tuberculosis and HIV. Despite the efforts to treat tuberculosis made by the Ivorian government, the incidence of the disease continues to grow [3]. Therefore, tuberculosis represents the leading opportunistic infection and cause of death among people infected with HIV living in the Ivory Coast. Multidrug-resistant tuberculosis remains a threat to the public health in the country and beyond the borders of Ivory Coast. Only one-third of individuals with drug-resistant tuberculosis received appropriate treatment in 2021 [4].

**Figure 1:**
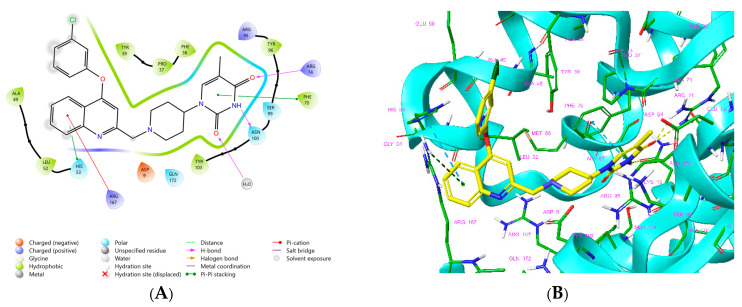

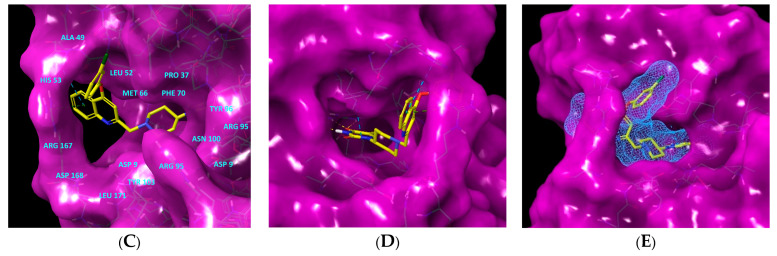
(**A**) Schematic drawing of compound **43** (TKI1) [10] at the *Mtb*TMK binding site showing residues in direct contact with the inhibitor [10,12]. (**B**) Three-dimensional structure of the *Mtb*TMK binding site with bound ligand compound **43** (${IC}_{50}^{exp}=$ 0.95 μM, ${IC}_{99}^{exp}\left(H37Ra\right)=12.17$ μM, PDB crystal structure 5NR7 [11]). The ligand and side chains of the interacting residues are represented as sticks. Carbon atoms are yellow (ligand) and green (enzyme), respectively. (**C**) Solid molecular surface of *Mtb*TMK modelled in Insight-II [15] shows the relatively large size of the entry into the thymidine binding cleft, which is partially filled by the *m*-chloro-phenoxyquinolin-2-yl group of the inhibitor **43**. (**D**) Another view of the binding cleft in the quinoline group direction. (**E**) The molecular surface (blue mesh) defines the volume occupied by the inhibitor **43**.

Tuberculosis is caused by *Mycobacterium tuberculosis* (*Mtb*), a bacterium that belongs to the genus *Mycobacterium*, the family *Mycobacteriaceae*, and the order *Actinomycetales*. *Mycobacteria* are strict or microaerophilic aerobic *bacilli* that are nonmotile and nonsporulating. They are characterized by a unique cell wall structure that confers specific staining properties and acid-alcohol resistance. Under optical microscopy, *Mtb* appears as a slender, slightly curved rod measuring 2 to 5 μm in length and 0.2 to 0.3 μm in width. It can be distinguished from other bacterial species by its specific culture requirements and slow growth rate [5]. The available antituberculotic agents face drug resistance from *Mycobacterium tuberculosis*, which can develop mechanisms to evade the antibacterial effect. The intrinsic resistance of *Mtb* to antibiotics is caused by a less permeable cell wall rich in lipids and the activity of efflux pumps that transport extrinsic substances out of the cell [6]. Multidrug-resistant tuberculosis (MDR-TB) refers to *Mtb* strains resistant to isoniazid and rifampicin, while extensively drug-resistant tuberculosis (XDR-TB) is also resistant to at least one of injectable antibiotics, such as capreomycin, kanamycin, or amikacin [7]. Treatment of drug-resistant forms of tuberculosis requires the development of new therapeutic alternatives. New antituberculotic agents should act on both active and dormant *Mtb* to prevent relapse of the disease during resuscitation of the bacteria to the growth phase [8]. Therefore, the identification of new potential pharmacological targets within the pathogen and the design of new bioactive compounds that act on these novel targets is urgently needed [1,9]. Among such targeted enzymes are those involved in vital processes, such as NAD supply and ATP phosphorylation. Drugs acting via novel mechanisms of action are preferred, as they are less amenable to drug cross-resistance with existing antituberculotics. Recently, the synthesis and testing of a new series of non-nucleoside inhibitors of *Mtb*TMK were reported [10,11,12]. The *Mtb*TMK is an essential enzyme involved in the biochemical pathway of deoxythymidine triphosphate synthesis. It catalyzes the conversion of deoxythymidine monophosphate to deoxythymidine diphosphate using ATP as a high-energy phosphate donor. The micromolar non-nucleoside thymidine analog **1** identified earlier [12] was further modified into three novel chemical series [10,12]. The first was represented by the phenoxylbenzyl analog **2,** the second truncated series was represented by the quinolin-2-yl analog **35**, and the third phenoxylquinolin-2-yl series was represented by derivative **43** (further denoted as TKI1) (Figure 1) with potent inhibitory activity toward *Mtb*TMK and an improved antibacterial MIC [10].

The availability of the crystal structure of *Mtb*TMK bound to deoxythymidine monophosphate (dTMP) [11] opened the possibility of designing new antituberculotic agents [13] based on theoretical approaches, such as molecular modelling and structure-based drug design, which can lead to the prediction of the binding mode and improve the binding affinity of new ligands to *Mtb*TMK. These computational approaches provide a means to describe intermolecular interactions and predict the inhibitory potency of new compounds based on calculated thermodynamic quantities, such as the free energy of drug–receptor complex formations [14]. The crystal structure of the *Mtb*TMK–TKI1 complex [11] showed that the entry into the thymidine binding cleft is relatively large and of a complex shape (Figure 1C–E). An extension of the *m*-chloro-phenoxy group and the quinolin-2-yl group of TKI1 by proper substituents filling parts of the entry could enhance the binding affinity as well as specificity of new TKI analogs towards the *Mtb*TMK.

In this work, we have performed computer-aided molecular design of new *Mtb*TMK inhibitors by in situ modification of the reference inhibitor **43** TKI1 [10] *5-methyl-1-[1-[(6-phenoxypyridin-2-yl)methyl]piperidin-4-yl]pyrimidine-2,4-dione* bound to the *Mtb*TMK of strain H37Rv (PDB entries: 5NRN and 5NR7 [10,11]) using the MM-PBSA approach [16]. We have explored the replacement of the *m*-chloro-phenoxyquinolin-2-yl group of inhibitor **43** studied by Song et al. [10], which fills the entry into the thymidine binding cleft of *Mtb*TMK lined with hydrophobic residues, using alternative larger hydrophobic groups. Our goal was to identify new non-nucleoside thymidine derivatives originating from compound **43** that display improved affinity and specificity of binding to a new protein target *Mtb*TMK with decreased cross-resistance with existing antituberculotics and show favorable ADME-related properties, especially elevated hydrophobicity, which improves permeation across the bacterial cell wall of *Mtb.* The presented new inhibitors could be developed as drugs candidates with antibacterial activity toward *Mtb* and could eventually lead to the creation of new antituberculotic medication.

## 2. Materials and Methods

**Scheme 1:**
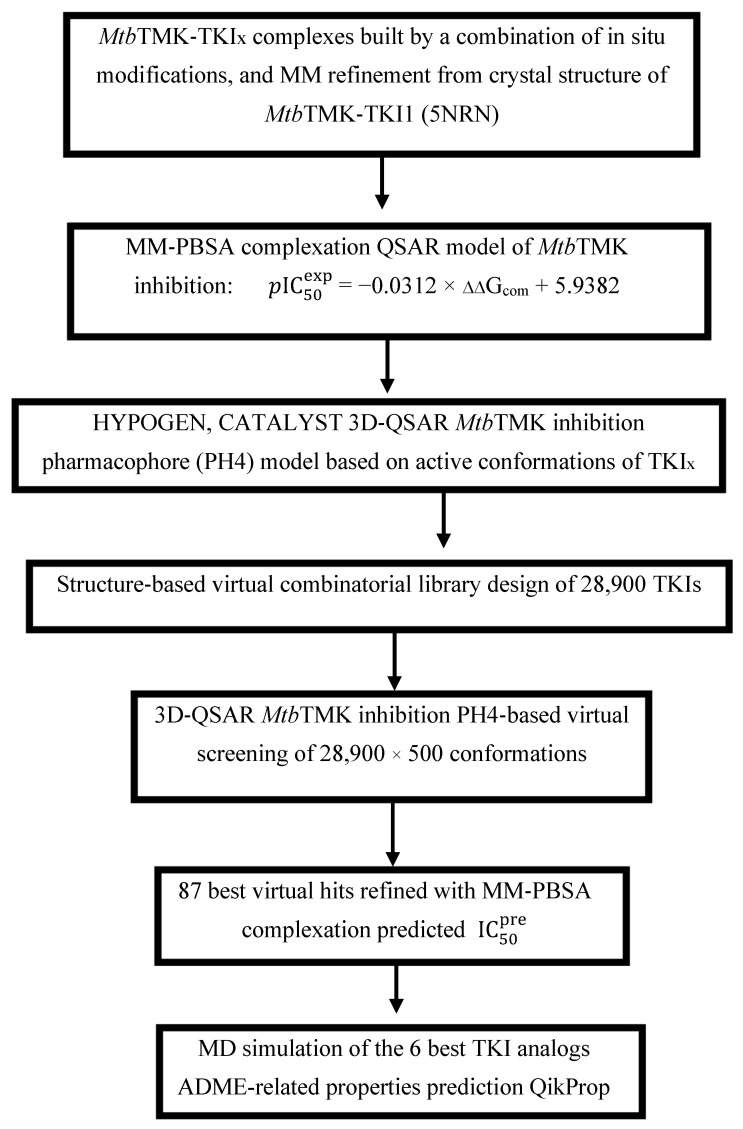
Workflow describing the sequence of steps taken to design new TKI analogs deriving from TKI1 [10] with higher predicted potencies towards *Mtb*TMK.

Scheme 1 presents the TKI discovery workflow outlining sequential steps of combinatorial ligand design and the in silico approach for generating new TKI analogs with enhanced affinity for *Mtb*TMK.

### 2.1. Training and Validation Sets

The chemical structures and observed inhibitory potencies (${IC}_{50}^{exp}$) of the training and validation sets of TKIs used in this study (Table 1) were taken from the literature [10,12]. The inhibitory potencies of these compounds span a wide range of half-maximal inhibitory concentrations (0.95 μM ≤ ${IC}_{50}^{exp}$ ≤ 2000 μM) which allows the construction of the QSAR model. The TS contains 31 TKIs and the VS contains 6 TKIs.

### 2.2. Model Building

The molecular models of the enzyme–inhibitor complexes *Mtb*TMK–TKIx, the apo-enzyme thymidylate kinase (E), and the unbound inhibitors (I) were generated based on the high-resolution crystal structure of the reference complex *Mtb*TMK–TKI1 obtained from the Protein Data Bank (PDB entry code 5NRN) at a resolution of 2.2 Å [10]. For this purpose, the Insight-II molecular modelling program [15] was used to build *Mtb*TMK–TKIx complexes for the inhibitors included in the training and validation sets by in situ structural modifications of TKI1.

To generate the TKI inhibitors included in TS and VS, the derivatized groups of the TKI1 moiety were replaced by appropriate substituents. A comprehensive conformational search was conducted for all rotatable bonds in the substituted functional group. This was followed by a gradual energy optimization of the modified inhibitor and the active site of *Mtb*TMK, which involved residues located within a 5 Å distance from the inhibitor. The goal of this process was to identify low-energy bound conformations of the modified inhibitors. The resulting structures of the enzyme–inhibitor complexes were further refined through protein-wide energy minimization. This methodology has previously been successfully used in the construction of models for viral, bacterial, and protozoal protease inhibitor complexes. It has also been used in the design of inhibitors based on peptidomimetic, hydroxyl naphthoic, and thymidine scaffolds [17,18,19].

The structures of the enzyme (E) and the enzyme–inhibitor complexes (E-Is) were considered at a physiologically relevant pH of 7. Neutral N- and C-terminal residues were capped, and all protonated and ionized residues were assigned appropriate formal and atomic charges. The crystallographic water molecules present in the reference complex *Mtb*TMK–TKI1 were excluded from the molecular models.

### 2.3. Molecular Mechanics

The inhibitors, thymidylate kinase of *Mtb*, and E-I complexes were modelled using molecular mechanics (MM) based on the previously described methodology [20,21,22]. Molecular mechanics was used to simulate the interactions between molecules. The systems were represented using a second-generation consistent force-field (CFF) [23,24], incorporating parameters, such as atomic charges and interatomic equilibrium distances. A CFF is a force field that accurately reproduces quantum mechanical energy surfaces, covers relatively large number of differing functional groups, is applicable in various phases and molecular environments, and is suitable for describing the interaction of protein active sites with different ligands or with solvents [23,24]. Therefore, the CFF approach is suitable for a study concerning a library of 9000 analogs.

Geometry optimization calculations were carried out to minimize the potential energy, employing methods, such as steepest descent and conjugate gradient. The dielectric constant was adjusted to reflect the protein environment, ensuring a realistic representation of molecular interactions.

### 2.4. Conformational Search

The free inhibitor conformations were derived from their bound configurations in the E-I complexes by gradual relaxation to the nearest local energy minimum as previously described [20,25,26,27]. The conformational search method involves systematically exploring the potential energy landscape of a molecular system to identify its stable conformations. This is achieved by varying dihedral and bond angles and bond lengths while employing energy minimization techniques to assess the stability of each conformation [20,25]. Various algorithms, such as Monte Carlo or simulated annealing, may be used to enhance sampling efficiency. The search parameters, including temperature, step size, and energy thresholds, are crucial for ensuring thorough exploration and convergence to low-energy conformations.

### 2.5. Solvation Gibbs Free Energies

The electrostatic component of Gibbs free energy (GFE) [28], which includes the effects of ionic strength by solving the nonlinear Poisson–Boltzmann equation [29,30], was computed by the DelPhi module in Discovery Studio [31] as previously described [16,29,30,32,33,34]. The Gibbs free energy of the solvation method quantifies the stability of molecules in solution by considering solute–solvent interactions. It relies on thermodynamic calculations and force field models to estimate enthalpic and entropic contributions, taking into account key parameters, such as the dielectric constant and temperature. The resulting free energies of solvation allow for the prediction of the solubility and biological activity of compounds in aqueous environments.

### 2.6. Entropic Contribution

The entropic contribution to the binding affinity of the enzyme–inhibitor complex was calculated by the normal mode analysis of the vibrational energy of the inhibitor at the active site of the ‘frozen’ enzyme and in the free state, as previously described [33].

The calculation of the entropic contribution involves assessing the configurational entropy of a molecular system, which reflects the number of accessible microstates. This is typically performed using statistical mechanics principles, such as the Boltzmann equation, to relate entropy to the probabilities of different configurations. Parameters, such as temperature, volume, and the nature of the molecular interactions, are essential for accurate calculations. Additionally, computational methods, like molecular dynamics or Monte Carlo simulations, can be employed to sample conformational space and obtain a more precise estimation of the entropic contribution.

### 2.7. PH4 Pharmacophore Generation

The PH4 pharmacophore model, derived from the bound TKI conformations at the Thymidylate kinase active site, was employed to query libraries, as reported earlier [21,35,36,37]. The generation of the pharmacophore PH4 involves identifying the spatial and electronic features essential for the biological activity of a compound. This process begins with the collection of data on active and inactive molecules, allowing for the extraction of common functional groups and structural motifs. Using computational tools, these features are arranged in three dimensions to create a pharmacophore model that reflects interactions with the target site. Key parameters, such as the types of interactions (hydrogen bonds, hydrophobicity) and their spatial orientations, are crucial for ensuring the accuracy and predictive power of the model in virtual screening applications.

### 2.8. ADME-Related Properties

The ADME properties from the bound TKI conformations at the Thymidylate kinase active site were employed to query libraries, as reported earlier [25,38,39,40]. ADME-related properties refer to the absorption, distribution, metabolism, and excretion characteristics of a compound, which are critical for evaluating its pharmacokinetic profile. This assessment involves computational and experimental methods to predict how a drug behaves in the body, including its permeability across biological membranes, plasma protein binding, metabolic stability, and clearance rates. Understanding these properties helps to optimize drug design and improve the efficacy and safety of pharmaceutical candidates.

### 2.9. Virtual Combinatorial Library

The virtual library was generated using a previously established protocol [20,27,41,42]. The generation of virtual libraries involves creating a diverse collection of molecular structures using the library design protocol in Discovery Studio 2.5 [31]. This process allows for the systematic exploration of chemical space by generating new compounds based on predefined scaffolds and functional groups. The resulting virtual library can then be screened for potential biological activity, thereby facilitating the identification of lead compounds for further development.

### 2.10. Inhibitory Potency Prediction

The conformer with the best mapping on the PH4 pharmacophore in each cluster of the focused library subset was used for ∆∆G_com_ calculation and ${IC}_{50}^{pre}$ estimation by the complexation QSAR model, as previously described [29]. The rGFE of formation of the E–I complex in water ∆∆G_com_ was computed for each selected new analog and then used to predict the *Mtb*TMK inhibitory potencies (${IC}_{50}^{exp}$) of the focused virtual library of TKI analogs by inserting this parameter into the target-specific scoring function. The scoring function, which is specific for the *Mtb* Thymidylate kinase receptor, is as follows: ${pIC}_{50}^{exp}$ = a × ∆∆G_com_+ b. It was parameterized using the QSAR model described in Section 3.1.

### 2.11. Molecular Dynamics

The conformational stability of the co-crystallized ligand (TKI1) and the five selected top-ranking designed TKI analogs (13_1, 13_4, 13_6, 149_5, 21_5) was examined by 200 ns-long MD simulations in NPT statistical ensemble (300 K, 1 bar) and analyzed using Desmond [43]. A periodic box with 10 Å buffer containing the *Mtb*TMK–TKI complex was filled with approx. 12,000 TIP3P water molecules and neutralized by adding ions to reach a state of electro-neutrality. During the simulation, an OPLS3e forcefield, 1.5 fs integration step, and coulombic interaction cut-off of 14 Å were used. After initial heating and relaxation, the productive simulation trajectory was recorded and analyzed for ligand–receptor interactions every 400 ps. Further details of the MD simulations are described in ref. [44].

## 3. Results and Discussion

### 3.1. QSAR Model of Thymidylate Kinase Inhibition

The training set (TS) of 31 and the validation set (VS) of 6 *Mtb*TMK inhibitors (TKI, Table 1) used in this study were selected from the literature [10,12]. The inhibitory potencies of these derivatives cover a sufficiently broad range of values (0.950 μM ≤ ${IC}_{50}^{exp}$ ≤ 2000 μM) to allow preparation of a QSAR model of *Mtb*TMK inhibition. Molecular mechanics (MM) was used to calculate the enzyme–inhibitor binding affinities.

For each of the 37 TKIs (Table 1), the enzyme–inhibitor complex *Mtb*TMK–TKIx was constructed by in situ modifications of the refined template crystal structure of the *Mtb*TMK–TKI1 complex (PDB entry code 5NRN [10,11]), as described in the Methods section. Furthermore, the relative Gibbs free energy values of the formation of the *Mtb*TMK–TKIx complex (∆∆G_com_) and its components were calculated for each geometry-optimized complex (Table 2). A QSAR model was prepared that correlates the computed ∆∆G_com_ with the experimental in vitro inhibitory potencies of TKIs ($p{IC}_{50}^{exp}\;$= −${l{og}_{10}IC}_{50}^{exp}$), which explained 95% of the variation in the observed TKI potencies and validated our molecular model of *Mtb*TMK inhibition for the subsequent design of new TKI analogs. The resulting linear regression equation is shown in Table 3 (Equation (2)). The relatively high values of the regression coefficient R^2^, leave-one-out cross-validated regression coefficient R^2^_xv_, and Fischer F-test of the correlation suggest a strong relationship between the 3D model of TKI binding and the observed inhibitory potencies. Therefore, structural information derived from the 3D models of the *Mtb*TMK–TKIx complexes can be expected to lead to a reliable prediction of the inhibitory potencies of *Mtb*TMK for new TKI analogs using Equation (2) (Table 3).

To gain a better structural understanding of the binding affinity of TKIs to *Mtb*TMK, we also analyzed the enthalpy of complex formation in the gas phase ∆∆H_MM_ by correlating it with the ${pIC}_{50}^{exp}$. The validity of the resulting linear correlation (for statistical data of this regression, see Table 3, Equation (1)) allowed the assessment of the contribution of enzyme–inhibitor interatomic interactions (∆∆H_MM_) to the ligand binding affinity when the solvent effect and loss of inhibitor entropy upon binding to the enzyme were neglected. This correlation explained about 89% of the ${pIC}_{50}^{exp}$ data variation and underlined the dominant role of alterations in the chemical structure of the ligands in improving the binding site interactions of the TKIs. These statistically significant correlations confirmed the correctness of the bound conformation of TKIs modelled at the *Mtb*TMK binding site and allowed the definition of TKI PH4 pharmacophore.

**Figure 2:**
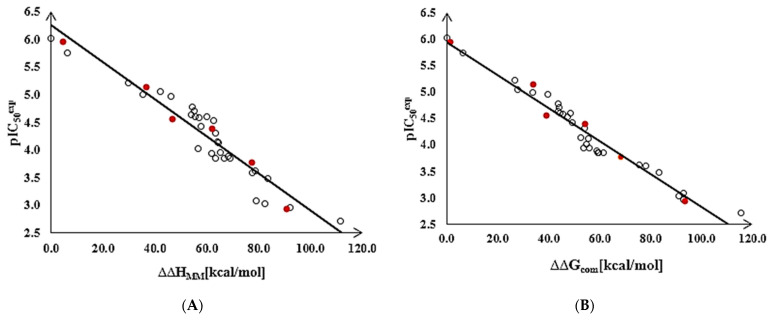
(**A**) Graph of the correlation Equation (1) (Table 3) between ${pIC}_{50}^{exp}$ [10] and the relative enthalpic contributions ∆∆H_MM_. (**B**) Graph of the correlation Equation (2) between ${pIC}_{50}^{exp}$ and the relative Gibbs free energies ∆∆G_com_ of the *Mtb*TMK–TKIx complex formation. Training set—open circles; validation set—red circles.

The statistical data confirmed the validity of the correlation Equations (1) and (2) shown in Figure 2. The ratio ${pIC}_{50}^{pre}$/${pIC}_{50}^{exp}$ ≈ 1 calculated for the validation set inhibitors TKIV1–TKIV6 documents the substantial predictive power of the QSAR model. The ${pIC}_{50}^{pre}$ values were estimated from the calculated ∆∆G_com_ using the correlation Equation (2), Table 3.

Thus, the validated QSAR model, regression equation Equation (2), and the calculated ∆∆G_com_ quantities of the *Mtb*TMK–TKIx complexes (Table 2) can be used to predict inhibitory potencies of the new analogs of TKI towards *Mtb*TMK, if they share the same binding mode as the TKI training set, which implies considering only the restricted modifications of the TKI scaffold.

### 3.2. Binding Mode of TKIs

Song et al. reported several comprehensive studies on the development of non-nucleoside inhibitors of *Mtb*TMK [10,11,12,17]. The thymine ring of these substrate-based TKI interacts with residues of the catalytic pocket in a configuration similar to the previously described crystal structure of dTMP bound to *Mtb*TMK and thymine-like inhibitors [10,17,18,19]. The thymine ring is stabilized by *π*-*π* stacking interaction [45,46], with Phe70 and hydrogen bonds linking the O4 and N3 of the thymine with Arg74 and Asn100, respectively [10,11,17]. The *p*-piperidine ring, modelled in a chair conformation, protrudes from the active site and is located in a position similar to the deoxyribose of dTMP [18], engaging in a *π*-alkyl interaction with the aromatic side chain of Tyr103 [45,46]. The phenoxypyridyl ring does not seem to establish any specific interaction with the enzyme except for the closure of the hydrophobic entry to the thymine pocket because of the solvent effect.

The binding mode of TKIs was adopted from the crystal structure 5NR7 [10,11], which contains the largest and most potent inhibitor TKI1 (**43**) [10]. Enzyme–inhibitor interactions at the active site of the *Mtb*TMK–TKI1 complex and the 3D structure of TKI1 at the kinase binding site are shown in Figure 1. One of the main contributions to the binding of TKI1 to the enzyme involves a hydrophobic pocket formed by the residues Phe36, His53, Phe70, Ala48, Ala60, Gly124, Asn100, Gly124, Arg153, Ala154, Gln155, Leu52, Arg107, Gly152, and Ser61 [10,12]. As can be seen in Figure 1, for inhibitor TKI1, the *m*-chloro-phenoxyquinolin-2-yl substituent reaches the hydrophobic entry to the binding cleft and contributes significantly to stabilizing the interactions at the active site of *Mtb*TMK. The introduction of new TKI1 analogs with a preserved binding mode to *Mtb*TMK and variation in the structure of the hydrophobic R-group can further enhance stabilizing enzyme–inhibitor interactions, resulting in the improved potency of the designed compounds. At the same time, the more hydrophobic character of TKIs can enhance the permeation of the lipid-rich *Mtb* cell wall and the antibacterial effect.

The inhibitor TKI1 with the largest R-group (Table 1), which fills the hydrophobic cavity at the entry of the thymidylate binding cleft, displayed the highest inhibitory potency to *Mtb*TMK. Therefore, we have explored the chemical space of larger hydrophobic groups by extending the *m*-chloro-phenoxyquinolin-2-yl group of the inhibitor **43** [10] at two other substitution sites.

### 3.3. D-QSAR Pharmacophore Model

**Figure 3:**
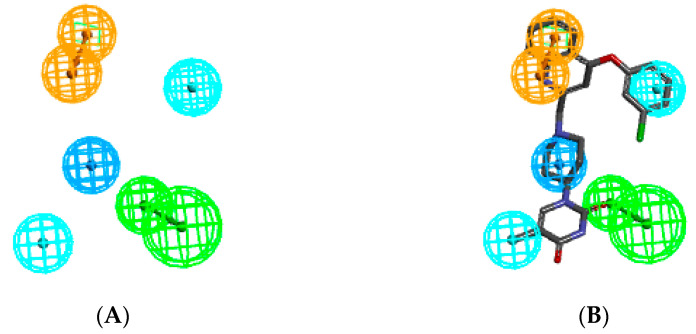

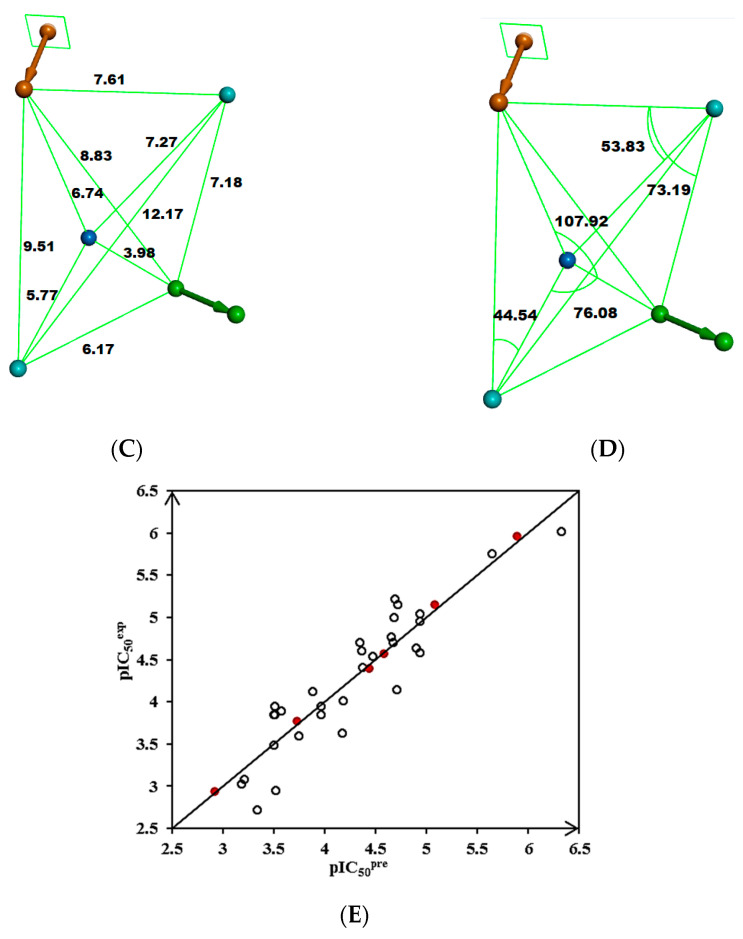
(**A**) Pharmacophore features, their centers, and vectors; HYDA = hydrophobic aliphatic (blue), HYD = hydrophobic (cyan), Ar = ring aromatic (orange), and HBA = hydrogen bond acceptor (green). (**B**) Features overlayed with inhibitor TKI1 (**43** [10]). (**C**) Distances between the centers of pharmacophoric features in [Å]. (**D**) Angles between the centers. The R-group of TKI1 is occupied by Ar and HYD features. (**E**) Correlation plot of ${pIC}_{50}^{exp}$ vs. ${pIC}_{50}^{pre}$ obtained for the PH4 pharmacophore model. Training set—open circles; validation set—red circles.

The ligand interaction protocol of Discovery Studio [31] generates receptor-based pharmacophore features of the active site of a protein–ligand complex. *Mtb*TMK predominantly exhibits hydrophobic features at the active site of a protein–ligand complex (Figure 3), as confirmed by previous studies [22,47]. The 3D-QSAR pharmacophore generation protocol, which uses the Catalyst HypoGen algorithm [15,31], was applied to construct the PH4 pharmacophore for *Mtb*TMK inhibition [22,47].

For each of the 10 hypotheses, a confidence level S of 98% was selected. The number of scrambled runs (Y) required to satisfy the relation S = [1 − (1 + X)/(1 + Y)] × 100%, where the number of hypotheses (X) with a total cost below that of Hypothesis 1 (Hypo1), was set at 0 (X = 0). Then, the total number of HypoGen runs (initial one + random runs) should be equal to 1 + Y = 50. The use of these values of X and Y results in the required S = 98%.

Validation of the statistical significance of each hypothesis model is carried out using Fisher’s randomization of the observed biological ${IC}_{50}^{exp}$ for the 31 training set compounds. It checks whether the PH4 model is not a result of an incidental correlation between ${IC}_{50}^{exp}$ and the properties of the TKI1–TKI31 samples (CatScramble program). From the lowest cost among the 49 hypotheses for each of the 10 PH4 (Hypo1–Hypo10) listed in Table 4 (closest random), none shows a cost lower than the 277.92 of Hypo1. Randomization confirmed that Hypo1 is not produced by chance and is a true correlation supported by a statistically robust pharmacophore model for the inhibition of *Mtb*TMK by TKI with a confidence level of 98%. The PH4 pharmacophore model shows predictive power comparable to the QSAR complexation model using the ligand binding affinity descriptor ∆∆G_com_ (R^2^ = 0.95, Table 3).

We will use the QSAR modes for the prediction of the inhibitory potencies ${IC}_{50}^{pre}$ of new TKI ligands which explore the hydrophobic substitutions of the *m*-chloro-phenoxyquinolin-2-yl group (the hydrophobic feature HYD, Figure 3B).

### 3.4. Virtual Combinatorial Library of TKIs

Our previous research on inhibitor design demonstrated that the use of in silico screening of a virtual combinatorial library can result in the identification of promising hits [20,22,47]. In a previous study focused on the antituberculotic effect of *Mtb*TMK inhibitors [20,22,41,48], we introduced a targeted virtual combinatorial subset. To do this, we carefully selected 40 diverse smaller polar and nonpolar, larger aliphatic, and aromatic hydrophobic building blocks for R_1_ of the TKI scaffold IV (Table 5). Additionally, for the R_2_ group, we also chose the same fragments as the R_1_ group that can be accommodated by the corresponding binding pocket of *Mtb*TMK.

To further explore structural variations in TKIs, we have prepared a virtual combinatorial library of new TKI analogs by extending the hydrophobic phenoxyquinolin-2-yl group of the most potent inhibitor TKI1 by substitutions at positions R_1_ and R_2_ (TKI scaffold IV in Table 5). The TKI scaffold IV is based on the published results of Song et al. [10], who showed that compounds of this type (**42**, **43**, and **44**) displayed potent inhibitory potencies toward *Mtb*TMK (${IC}_{50}^{exp}$ from 0.95 to 1.8 µM) [10]. The R-groups taken from Available Chemicals Directory [49] listed in Table 5 were attached to the R_1_ and R_2_ positions of the TKI scaffold IV and formed a combinatorial library of R_1_ × R_2_ = 170 × 170 = 28,900 TKI analogs. This virtual library was generated with the help of the enumerate library module of Discovery Studio [31].

To identify new TKIs with a strong binding affinity to the *Mtb*TMK target, the virtual combinatorial library of 28,900 TKI analogs was subjected to in silico screening against the PH4 pharmacophore model. During the virtual screening, 1000 conformers were generated for each analog. As a result of the screening, 10,498 TKIs were found to match at least 2 pharmacophoric features of the Hypo1 hypothesis of the PH4 pharmacophore model of *Mtb*TMK inhibition (Figure 3A,B). These matching analogs, known as PH4 hits, were then subjected to further evaluation using the complexation QSAR model. The complexation model used the relative Gibbs free energy of the formation of the *Mtb*TMK–TKIx complex and predicted corresponding ${IC}_{50}^{pre}$ (Table 6) values using correlation Equation (2) (Table 3).

### 3.5. New Inhibitors of MtbTMK

The findings of the in silico screening of the virtual library of novel TKI analogs were further investigated by analyzing the frequency of occurrence of each individual fragment within the R-groups of the 10,498 virtual hits that matched the PH4 model. Analysis revealed that R_1_ substituents, which occupy the hydrophobic pocket associated with the HYD pharmacophoric feature (*m*-chloro-phenoxy group in TKI1), play a dominant role in increasing the potency of new TKIs.

We have predicted half-maximal inhibitory concentrations for a series of the most promising new TKI analogs by calculating the binding affinity (∆∆G_com_) to *Mtb*TMK using the MM/PBSA approach [16] and estimated the corresponding ${IC}_{50}^{pre}$ from Equation (2) (Table 3). The results are shown in Table 6. The predicted ${IC}_{50}^{pre}$ values of the best new TKIs obtained from the QSAR model (Table 3) rest in the low nanomolear concentration range and exceed the range of experimental activity values of training and validation sets of TKI [10,12]. Therefore, we do not consider the ${IC}_{50}^{pre}$ exact but rather a strong indication that further extension of the phenoxyquinolin-2-yl group of TKI by suitable predominantly hydrophobic substituents in positions R_1_ and R_2_ leads to more potent inhibitors of *Mtb*TMK than the best reference compound **43** (TKI1) [10]. The calculated ${IC}_{50}^{pre}$ values help us to pick the appropriate substituents.

**Figure 4:**
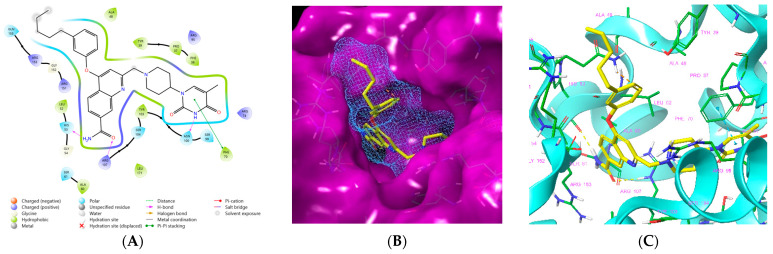
(**A**) Two-dimensional interaction diagram of the most active TKI analog 149_5. (**B**) Molecular volume of the hydrophobic substituted *m*R_2_ -phenoxy-7R_1_-quinolin-2-yl group. (**C**) Three-dimensional structure of *Mtb*TMK binding site with the bound TKI analog 149_5.

One of the most active TKI analogs 5_149 ($\;{IC}_{50}^{pre}$ = 15.5 nM, Table 6) contains *m*-pentyl-phenoxy and 7-formamide-quinolin-2-yl groups that occupy the entry into the thymidine-binding cleft of *Mtb*TMK. The R_2_ pentyl chain (5, Table 5) makes hydrophobic contact with the side chains of GLN155 and ARG151, the R_1_ formamide (149) group cross-links the His53 and Arg107 residues with 2 hydrogen bonds, while the thymidine building block of the analog essentially preserves the binding mode seen in the crystal structure of the *Mtb*TMK–TKI1 complex [10] (Figure 4). The main improvement in the predicted Gibbs free energy of *Mtb*TMK–TKI_5_149 complex formation ∆∆G_com_ comes from the enthalpic contribution of ∆∆H_MM_, which includes the formation of additional two hydrogen bonds.

Analysis of the computational results shown in Table 6 suggests that attachment of various R_1_ and R_2_ groups to the TKI scaffold IV (Table 5) leads to enhanced predicted binding affinity of analogs the *Mtb*TMK compared to the reference inhibitor TKI1 [10]. However, the most promising new TKI analogs 5_149, 13_4, 13_6, 13_1, 149_5, and 5_21 contain predominantly aliphatic chains in the R_1_ position and shorter polar structures capable of hydrogen bond formation in the R_2_ position. The predicted activity ${IC}_{50}^{pre}$ of the best new TKI 5_149 is more than 60 times lower than that of the reference inhibitor TKI1 [10]. It is, therefore, worth exploring the further derivatization of the TKI scaffold IV in the R_1_ and R_2_ positions when development of more potent antituberculotic agents is desired. The calculated Gibbs free energies of complex formation (Table 6) suggest that the best designed analogs will bind the *Mtb*TMK more strongly than the reference compound **43** (TKI1) [10] and exert a higher pharmacodynamic effect.

### 3.6. ADME-Related Properties of New TKI Analogs

The pharmacokinetic profile of the new TKI analogs was predicted using the QikProp software (version 6.5) [38,39,50] (Table 7) and used for the selection of drug-like inhibitor candidates [22,40,47]. The 10 new TKI analogs included in Table 7 show favorable pharmacokinetic profiles, as documented by the global descriptor #starts, which indicates the number of ADME-related descriptors that fall outside the optimal range of values obeyed by 95% of known drugs out of the 24 calculated main QikProp descriptors [38,39,50]. The ADME properties of the TKI analogs are more promising than most of the reference antibiotics included in Table 7, especially in terms of lipophilicity and oral bioavailability.

The addition of the R_1_ and R_2_ groups to TKI1 affects not only ligand binding but also the solubility, metabolic stability, and other ADME-related properties of the new analogs. It is worth mentioning that of the six most promising new TKI analogs 5_149, 13_4, 13_6, 13_1, and 5_21, all compounds display a predicted #stars parameter equal to 0 (Table 7). This global drug-likeness descriptor shows that the new TKIs are predicted to possess favorable ADME properties and represent drug-like molecules. Furthermore, the enhanced hydrophobic character of the new TKIs (elevated hydrophobic molecular surface area S_mol,hfo_ as compared to TKI1, Table 7) can help increase the permeation of the inhibitors across the lipid-rich *Mtb* cell wall and facilitate the antibacterial effect.

### 3.7. Molecular Dynamics Simulations

**Figure 5:**
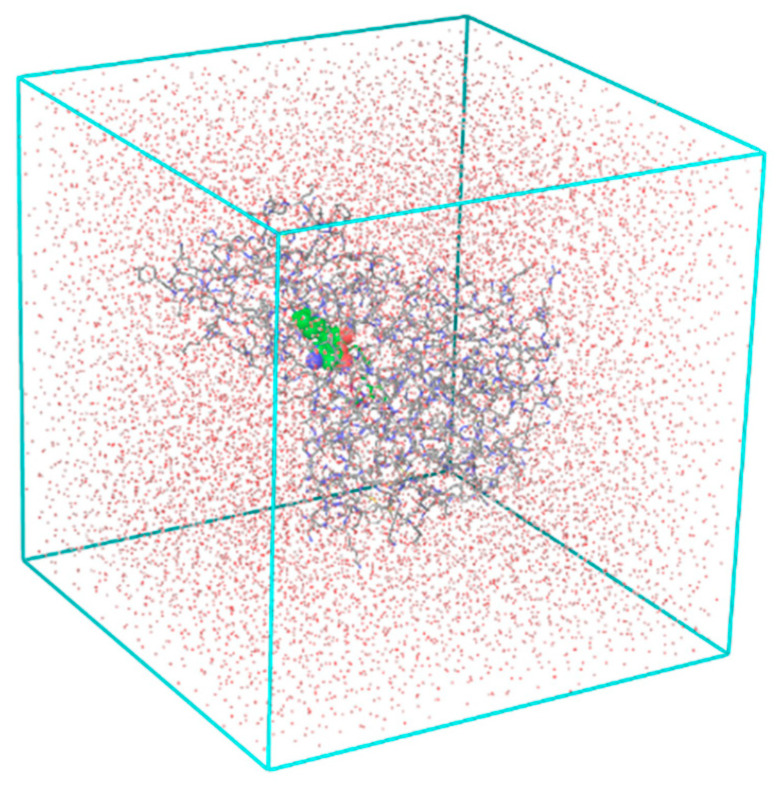
The periodic cubic box used in the MD simulations contains a solvated *Mtb*TMK–TKI1 complex.

**Figure 6:**
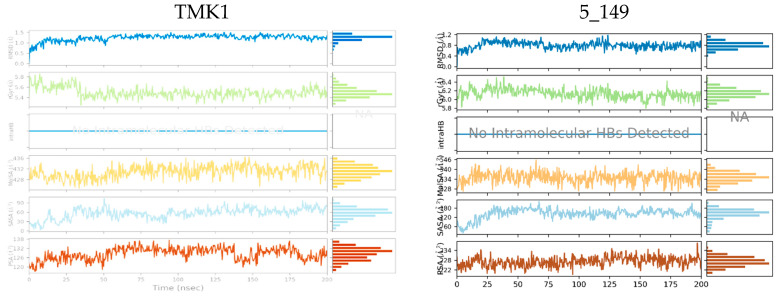

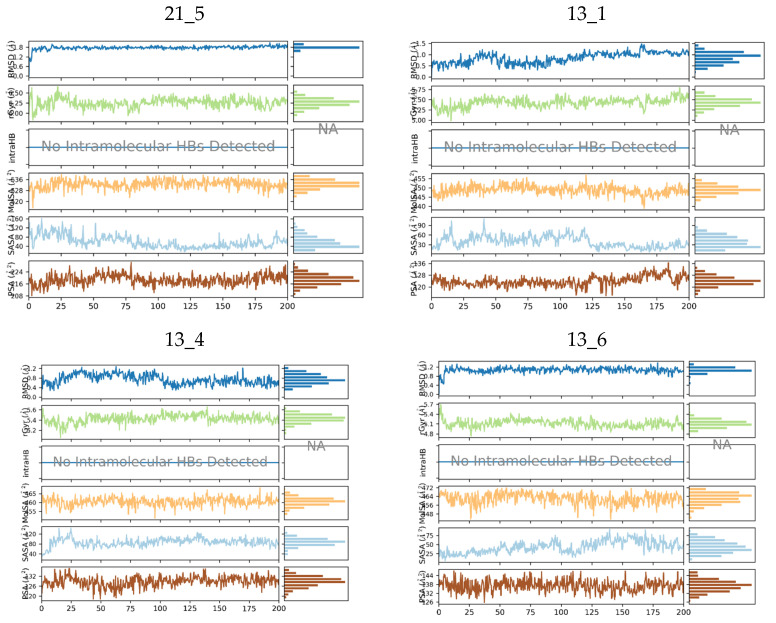
Molecular properties of six TKIs (Table 8) bound to *Mtb*TMK were monitored over 200 ns of MD simulation time. For each inhibitor root mean square deviation (RMSD) with respect to the initial conformation of the TKI, the radius of gyration (rGyr), number of intramolecular hydrogen bonds (intraHB), molecular surface area (molSA), solvent-accessible surface area (SASA), and polar surface area (PSA) were plotted (top to bottom in each diagram).

We have performed molecular dynamics (MD) simulations to check the stability of *Mtb*TMK–TKIx complexes and the flexibility of the bound conformations at the active site of *Mtb*TMK for the native ligand TKI1[10] and five best designed TKI analogs. The structures of the complexes obtained from combinatorial design approach and subsequent MM refinement were used as starting geometries for MD simulations using Desmond [43,44]. Figure 5 shows the periodic box with a solvated *Mtb*TMK–TKI1 complex. The ensemble averages of the total and potential energy of *Mtb*TMK–TKIx complexes for the TKI1 and the best new inhibitor candidates are shown in Table 8. Figure 6 shows the time evolution of the properties of the bound inhibitors, which documents that the inhibitors remain in their bound conformations, while their pose is slightly affected by thermal fluctuations.

**Figure 7:**
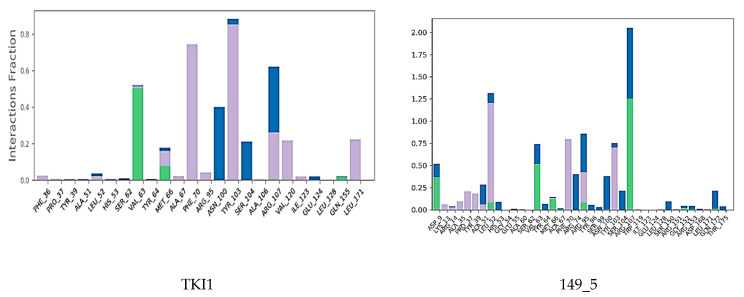

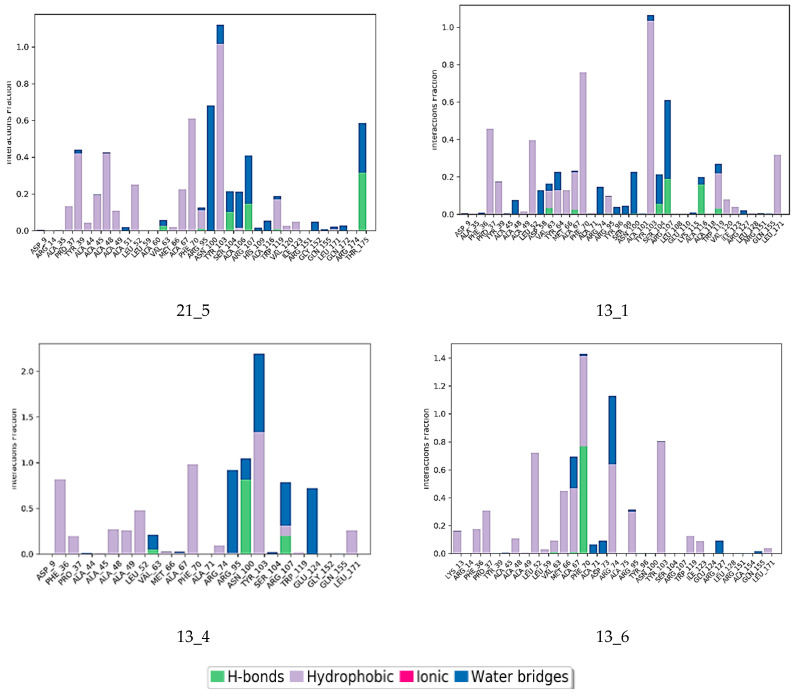
Contribution of individual active site residues to inhibitor binding in *Mtb*TMK–TKIx complexes present during at least 20% of the MD simulation time.

**Figure 8:**
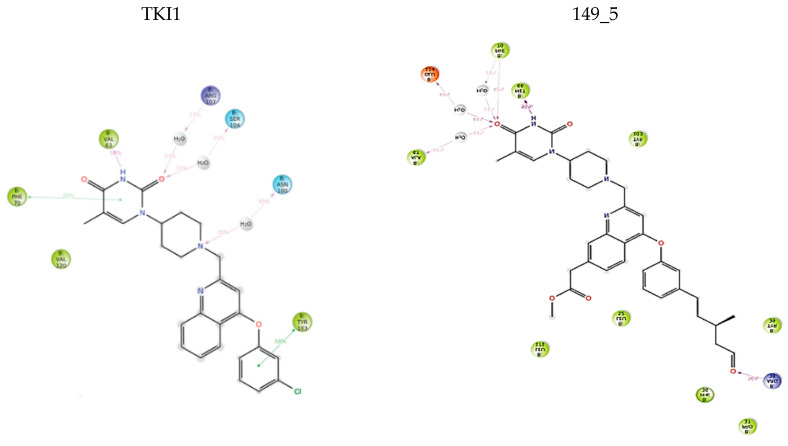

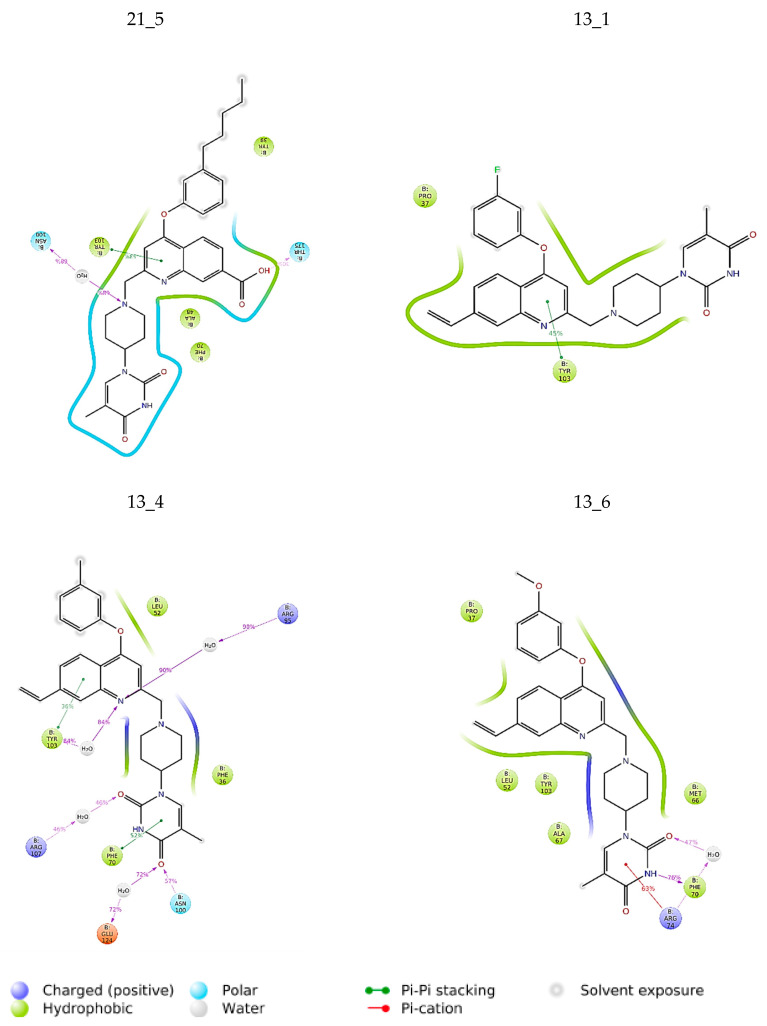
The 2D scheme presents attractive interactions prevalent between the functional groups of the six inhibitor candidates and the individual residues of the active site of *Mtb*TMK. These interactions were observed in at least 20% of the analyzed 500 frames of the MD trajectory (160 ns, 400 frames).

**Figure 9:**
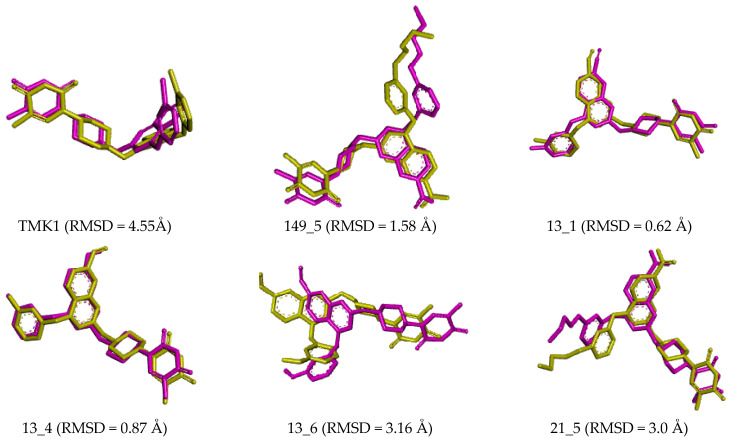
Overlay of the averaged bound conformation of the TKI from MD simulation (purple color) and MM-refined complexes (yellow color).

Interactions between the protein and TKI were observed throughout the MD trajectory to identify specific residue–inhibitor interactions maintained during the time evolution (Figure 7). Hydrophobic interactions seem to dominate the TKI1 binding to *Mtb*TMK. The new TKI analogs show higher contribution of hydrogen bonding and polar interactions with active site residues compared to TKI1, which indicates an increase in specificity of these inhibitor candidates for the *Mtb*TMK target. Finally, the interactions that occur over more than 20.0% of the simulation time are plotted in a 2D schematic representation (Figure 8). Moreover, we have superimposed averaged conformations of ligands obtained from MD simulation after MM minimization on those prepared by in situ modification and MM refinement of the TKI1 [10]. Figure 9 illustrates these superimposed structures and gives the respective RMSD.

Small deviations observed between the superimposed active conformations of new *Mtb*TMK inhibitors (except 14_115 and 5_58), which were modelled by in situ modification of TKI1 and MM-PBSA refinement, and those derived from averaging the MD simulation snapshots over the simulation time interval (Figure 9), indicate the stability of the modelled *Mtb*TMK–TKIx complexes. MD simulations preserved the binding mode of the TKIs observed in the crystal structure of *Mtb*TMK complexes [10,11,12].

## 4. Conclusions

In this study, new derivatives of TKI that are predicted to inhibit *Mtb*TMK at low nanomolar inhibitory concentrations were investigated (Table 8). A PH4 pharmacophore model of *Mtb*TMK inhibition, prepared with the help of a training set of 31 TKIs, was used to screen in silico a virtual combinatorial library consisting of more than 28,900 new TKI analogs. More than 89 top-scoring virtual hits were docked into the active site of *Mtb*TMK adopted from the crystal structure of the *Mtb*TMK–TKI1 complex [11,12,17,18,19]. The inhibitory potency of the designed compounds toward *Mtb*TMK was predicted based on the relative Gibbs free energies ∆∆G_com_ calculated for the formation of the enzyme–inhibitor complex (Table 6). In silico screening of the virtual library based on the match with the PH4 pharmacophore helped to identify appropriate R_1_ and R_2_ groups from the 170 substituents considered (Table 5) which are recommended for their derivatization of the TKI scaffold IV. Most of the 89 promising new TKI analogs (Table 6) with the hydrophobic *m*-chloro-phenoxyquinolin-2-yl group extended at positions R_1_ and R_2_ were predicted to inhibit the *Mtb*TMK better than the reference inhibitor TKI1 (**43**) (${IC}_{50}^{exp}=$ 950 nM) [10]. These novel TKI analogs, namely 4_19 (${IC}_{50}^{pre}$ = 41.1 nM), 115_14 (${IC}_{50}^{pre}$ = 17.2 nM), 149_5 (${IC}_{50}^{pre}$ = 15.5 nM), 13_1 (${IC}_{50}^{pre}$ = 9.5 nM), 13_4 (${IC}_{50}^{pre}$ = 9.9 nM), 13_6 (${IC}_{50}^{pre}$ = 10.8 nM) and 21_5 (${IC}_{50}^{pre}$ = 10.1 nM), 115_14 (${IC}_{50}^{pre}$ = 17.2 nM), and 46_16 (${IC}_{50}^{pre}$ = 26.2 nM), exhibit strong predicted binding affinities to *Mtb*TMK, (Table 6) in the low nanomolar concentrations range (TKI 13_1 is predicted to be 100 times more potent than TKI1) and possess favorable ADME-related properties (Table 7). Although our predictions of inhibitory potencies might be somewhat too optimistic, they clearly suggest that further extension of the hydrophobic cap of the *Mtb*TMK active site by the addition of small polar and nonpolar R_1_ and R_2_ groups to TKI is useful and leads to further improvement of binding affinity.

Thus, these novel derivatives show potential as possible candidates for reversible inhibitors that inactivate the proven pharmacological target *Mtb*TMK. This target is different from those related to the drug resistance of *Mycobacterium tuberculosis* to conventional antituberculotics. Molecular dynamics simulations confirmed stable enzyme–inhibitor complexes for these new inhibitor candidates. In conclusion, we suggest the synthesizing and testing of these new molecules to assess their inhibitory capabilities and their eventual development into potential antitubercular agents.

## Figures and Tables

**Table 1:** Training set and validation set of *Mtb*TMK inhibitors (TKI) used for the preparation of QSAR models of the enzyme inhibition. The experimental activities ${IC}_{50}^{exp}$ were taken from references [10,12].

TKI Scaffolds [10]							
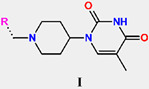			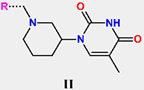			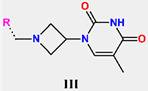	
TKI Scaffold	Training Set Inhibitor	R-Group	${IC}_{50}^{exp}$ [µM]	TKI Scaffold	Training Set Inhibitor	R-Group	${IC}_{50}^{exp}$ [µM]
**I**	TKI1	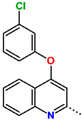	0.95	**I**	TKI10	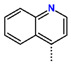	25
**I**	TKI2	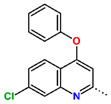	1.8	**I**	TKI11	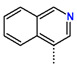	25
**I**	TKI3	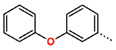	6.1	**I**	TKI12	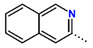	26
**I**	TKI4	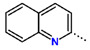	9	**I**	TKI13	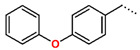	27
**II**	TKI5	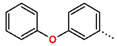	10	**I**	TKI14	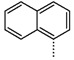	38
**I**	TKI6	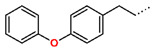	11	**I**	TKI15	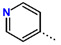	49
**I**	TKI7	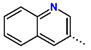	17	**I**	TKI16	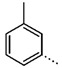	72
**I**	TKI8	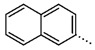	20	**I**	TKI17	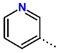	75
**I**	TKI9	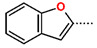	23	**I**	TKI18	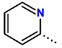	96
**I**	TKI25	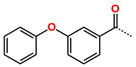	235	**I**	TKI19	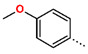	113
**I**	TKI26	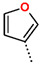	254	**I**	TKI20	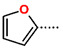	114
**I**	TKI27	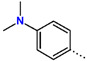	328	**I**	TKI21	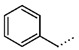	129
**III**	TKI28	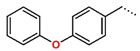	832	**I**	TKI22	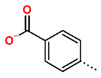	141
**III**	TKI29	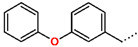	944	**I**	TKI23	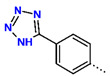	142
**I**	TKI30	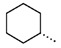	1115	**I**	TKI24	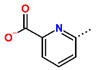	143
**III**	TKI31	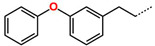	1910				
**TKI scaffold**	**Validation set inhibitor**	**R-group**	${IC}_{50}^{exp}$ **[µM]**	**TKI scaffold**	**Validation set inhibitor**	**R-group**	${IC}_{50}^{exp}$ **[µM]**
**I**	TKIV1	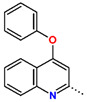	1.1	**I**	TKIV4	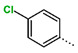	40.
**I**	TKIV2	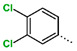	7.1	**I**	TKIV5	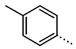	168
**I**	TKIV3	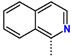	27	**III**	TKIV6	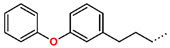	1142

**Table 2:** Relative complexation Gibbs free energy and its components for the training set of the *Mtb*TMK inhibitors TKI1–TKI31 and the validation set inhibitors TKIV1–TKIV6.

Training Set ^a^	∆∆H_MM_^b^ [kcal/mol]	∆∆G_sol_^c^ [kcal/mol]	∆∆TS_vib_^d^ [kcal/mol]	∆∆G_com_^e^ [kcal/mol]	${IC}_{50}^{exp}$ ^f^ [μM]
TKI1	0	0	0	0	0.95
TKI2	6.4	−0.5	−0.6	6.5	1.8
TKI3	29.6	1.6	4.4	26.7	6.1
TKI4	42.0	−7.7	6.5	27.8	9
TKI5	35.5	3.67	5.5	33.7	10
TKI6	46.1	−0.3	6.1	39.7	11
TKI7	54.4	−4.4	6.4	43.6	17
TKI8	55.1	−4.3	6.9	44.0	20
TKI9	54.0	−4.8	5.4	43.9	23
TKI10	60.0	−4.5	6.8	48.6	25
TKI11	55.4	−3.6	7.3	44.5	25
TKI12	56.8	−4.9	6.5	45.5	26
TKI13	62.5	−8.9	6.0	47.6	29
TKI14	57.7	−1.1	7.2	49.4	38
TKI15	63.2	−4.6	4.5	54.2	49
TKI16	64.0	−4.3	7.0	52.7	72
TKI17	64.6	−4.2	4.8	55.6	75
TKI18	56.6	1.4	3.2	54.8	96
TKI19	65.2	−5.2	6.4	53.6	113
TKI20	61.9	−1.3	4.6	56.0	114
TKI21	68.1	−3.9	5.4	58.8	129
TKI22	66.6	−2.6	4.9	59.1	141
TKI23	68.8	−4.5	4.7	59.6	142
TKI24	63.5	3.0	5.0	61.5	143
TKI25	78.5	0.2	3.1	75.6	235
TKI26	77.6	5.3	4.8	78.1	254
TKI27	83.4	8.7	8.9	83.1	328
TKI28	79.0	13.1	−1.0	93.0	832
TKI29	82.3	13.1	4.4	91.0	944
TKI30	92.2	11.8	11.3	92.7	1115
TKI31	111.5	13.3	9.4	115.4	1910
**Validation set ^a^**	**∆∆H_MM_^b^** **[kcal/mol]**	**∆∆G_sol_^c^** **[kcal/mol]**	**∆∆TS_vib_^d^** **[kcal/mol]**	**∆∆G_com_^e^** **[kcal/mol]**	${pIC}_{50}^{pre}$**/**${pIC}_{50}^{exp}$ **^g^**
TKIV1	4.2	−1.9	0.9	1.4	0.99
TKIV2	36.4	−0.1	2.5	33.7	0.95
TKIV3	46.4	−0.7	6.8	38.9	1.03
TKIV4	61.7	−3.9	3.8	54.0	0.97
TKIV5	77.3	−3.0	6.2	68.1	1.01
TKIV6	90.6	6.9	4.3	93.2	1.03

^a^ For chemical structures of the TKI analogs of TS and VS, see Table 1; ^b^ ∆∆H_MM_ is the relative enthalpic contribution to the change in ∆∆G_com_ in the formation of the enzyme–inhibitor E:I complex formation derived from molecular mechanics (MM): ∆∆H_MM_ = [E_MM_{E-I_x_}-E_MM_{I_x_}]-[E_MM_{E:I_ref_}-E_MM_{I_ref_}], I_ref_ is the reference inhibitor TKI1. ^c^ ∆∆G_sol_ is the solvent effect contribution to the ∆∆G_com_ change in the E:I complex formation: ∆∆G_sol_ = [G_sol_{E:I_x_}-G_sol_{I_x_}]-[G_sol_{E:I_ref_}-G_sol_{I_ref_}]. ^d^ ∆∆TS_vib_ is the relative entropic contribution to the ∆∆G_com_ of E:I complex formation: ∆∆TS_vib_ = [TS_vib_{I_x_}_E_-TS_vib_{I_x_}]-[TS_vib_{I_ref_}_E_-TS_vib_{I_ref_}]. ^e^ ∆∆G_com_ is the relative Gibbs free energy contribution change in the E:I complex formation: ∆∆G_com_ = ∆∆H_MM_ + ∆∆G_sol_-∆∆TS_vib_. ^f^ ${IC}_{50}^{exp}$ is the experimental half-maximal inhibitory concentration of TKI against *Mtb*TMK obtained from the literature [10,12]. ^g^ The ratio of predicted and experimental half-maximal inhibitory concentrations ${pIC}_{50}^{exp}$/${pIC}_{50}^{exp}$ was predicted from the computed ∆∆G_com_ using the regression Equation (2) shown in Table 3.

**Table 3:** Regression analysis of computed binding affinities ∆∆G_com_, its enthalpic component ∆∆H_MM_, and experimental half-maximal inhibitory concentrations ${pIC}_{50}^{exp}$ = −${l{og}_{10}IC}_{50}^{exp}$ of TKIs towards *Mtb*TMK [10,12].

Statistical Data of Regression	Equation (1)	Equation (2)
${pIC}_{50}^{exp}\;$= −0.0335 × ∆∆H_MM_ + 6.2453 (1) ${pIC}_{50}^{exp}\;$= −0.0312 × ∆∆G_com_ + 5.9382 (2)		
Number of compounds in TS	31	31
Squared correlation coefficient of regression R^2^	0.90	0.95
Cross-validated squared correlation coefficient R^2^_xv_	0.89	0.95
Standard error of regression σ	0.24	0.16
Statistical significance of regression, Fisher F-test	265.97	612.29
Level of statistical significance α	>95%	>95%
Range of experimental activities ${IC}_{50}^{exp}$ [μM]	0.95–1910	

**Table 4:** Parameters of ten generated PH4 pharmacophoric hypotheses for the *Mtb*TMK inhibitor after passing the CatScramble validation procedure. Each hypothesis was subjected to 49 random runs at a selected confidence level of 98%.

Hypothesis	RMSD ^a^	R^2 b^	Total Cost ^c^	Costs Difference ^d^	Closest Random ^e^	Features ^f^
Hypo1	3.34	0.92	277.92	1058.5	634.07	HBA, HYD-AL, HYD-AL, HYD, Ar
Hypo2	3.46	0.91	292.90	1043.5	657.28	HBA, HYD-AL, HYD, HYD, Ar
Hypo3	3.47	0.91	293.85	1042.6	670.39	HBA, HYD-AL, HYD, HYD, Ar
Hypo4	3.49	0.91	296.58	1039.8	673.41	HBA, HYD-AL, HYD, HYD, Ar
Hypo5	3.50	0.91	297.17	1039.3	683.53	Ar, HBA, HYD-AL, HYD-AL, HYD
Hypo6	3.51	0.91	299.60	1036.8	686.07	HBA, HYD, HYD, HYD, Ar
Hypo7	3.56	0.90	304.88	1031.5	695.91	HBA, HYD-AL, HYD, HYD, Ar
Hypo8	3.64	0.90	315.45	1021.0	709.05	HBA, HYD-AL, HYD, HYD, Ar
Hypo9	3.67	0.90	319.53	1016.9	744.34	HBA, HYD-AL, HYD, HYD, Ar
Hypo10	3.37	0.90	324.07	1012.4	747.03	HBA, HYD, HYD, HYD, Ar
Fixed cost	0	1	82.55			
Null cost	0	0	1336.43			

^a^ Root mean square deviation [Å]; ^b^ squared correlation coefficient; ^c^ overall cost parameter of the PH4 pharmacophore; ^d^ cost difference between the null cost and total cost of this hypothesis; ^e^ lowest cost of 49 scrambled runs at a selected level of confidence of 98%. Fixed cost = 82.55 with RMSD = 0, null cost = 1336.43 with RMSD = 8.53, and configuration cost = 18.89. ^f^ HBA (hydrogen-bond acceptor); HYD (hydrophobic); HYD-AL (hydrophobic aliphatic); HYD-Ar (hydrophobic aromatic); Ar (ring-aromatic).

**Table 5:** List of R_1_ and R_2_ groups used in the design of the initial diversity library of extended TKI analogs.

TKI Scaffold IV		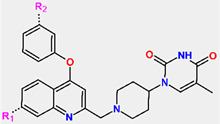			
		R_1_ and R_2_ Groups			
1	Fluoro	2	Chloro	3	propyl
4	Methyl	5	Pentyl	6	Methoxy
7	Isobutyl	8	Propylaldehyde	9	Heptylaldehyde
10	butylaldehyde	11	Pentylaldehyde	12	Acetaldehyde
13	Ethyl-1-ene	14	3-methylpentanal	15	3-phenylpropanal
16	3-methylbutanal	17	Propyl-1,2-diene	18	Propyl-1-ene
19	Formaldehyde	20	Benzaldehyde	21	Formic acid
22	3-(1H-pyrrol-2-yl)propanal	23	Hydroxyl	24	Ethenone
25	Ethenethione	26	Phenyl	27	Cyclohexyl
28	4-methylphenyl	29	4-ethylphenyl	30	3-fluorophenyl
31	4-fluorophenyl	32	m-3-hydroxyphenyl	33	p-3-hydroxyphenyl
34	3,5-dihydroxyphenyl Thiopyran	35	Thiopyran-3-yl	36	Thiopyran-4-yl
37	Methylbenzene	38	p-xylene	39	1-(fluoromethyl)-3-methylbenzene
40	1-(fluoromethyl)-4-methylbenzene	41	m-methylphenol	42	p-methylphenol
43	methyl-4-methoxybenzene	44	6-methylpyridine-3-thiol	45	4-methylbenzenethiol
46	4-methyl-2H-pyran	47	2,5-dimethylfuran	48	2-methylthiophene
49	2,5-dimethylthiophene	50	2,3-dimethylthiophene	51	2-thienyl
52	2-methylthienyl	53	3-methylthienyl	54	Furyl
55	3-methylfuryl	56	phenylamide	57	p-benzoyl acid
58	m-benzoyl acid	59	Cyclopenta-1,3-diene-5-yl	60	3-methylcyclopenta-1,3-diene-5-yl
61	3-ethylcyclopenta-1,3-diene-5-yl	62	3-methoxycyclopenta-1,3-diene-5-yl	63	2-methyloxazole
64	2-methylthiazole	65	2-methylimidazole	66	m-3-methylcyclopenta-1,4-dienol
67	4-methylcyclopenta-2,5-diene-1,2-diol	68	p-3-methylcyclopenta-1,4-dienol	69	cyclopenta-1,4-dienol-3-yl
70	cyclopenta-2,5-diene-1,2-diol-4-yl	71	methylcyclopropane	72	3-methylcycloprop-1-ene
73	1-methyl-1H-oxiren-1-ium	74	1-methyl-1H-thiiren-1-ium	75	1-methyl-1H-azirine
76	1-(methyl-2,3-dihydro-1H-imidazole	77	4-methylfuryl	78	2-ethyloxazole
79	ethyl	80	1-chloro-3-methylbenzene	81	(E)-vinylbenzene
82	2-chlorophenyl	83	(Z)-5-vinylcyclopenta-1,3-diene	84	2,5-dichlorophenyl
85	(E)-5-vinylcyclopenta-1,3-diene	86	3-chloro-5-fluorophenyl	87	(Z)-2-vinyl-1,3-dioxole
88	3,4-dimethylfuran-2-yl	89	Pyrimidin-2-yl	90	Pyridin-2-yl
91	2-methylpyridine	92	2-methylpyrimidine	93	3-methylisoxazole
94	5-bromophenyl	95	3-bromophenyl	96	3-bromo-1-methylbenzene
97	Isopentyl	98	4-bromo-1-methylbenzene	99	3,5-dibromo-1-methylbenzene
100	4-chloropyridin-5-yl	101	2-chloro-5-methylpyridine	102	3-chloro-5-methylpyridine
103	5-methylpyridin-3-yl	104	3,5-dimethylpyridine	105	1,2-dimethylcyclopropane
106	1,3-dimethylcycloprop-1-ene	107	1,2-dimethyl-1H-azirine	108	1,2,3-trimethylcycloprop-1-ene
109	1-ethylcycloprop-1-ene-3-yl	110	1-ethyl-2-methylcycloprop-1-ene-3-yl	111	1,2-diethylcycloprop-1-ene-3-yl
112	cycloprop-1-enol-3-yl	113	azirin-2-ol-1-yl	114	azirin-2,3-diol-1-yl
115	methyl acetate	116	Methyl formate	117	methyl 2-(formyloxy)acetate
118	N-hydrosulfonylformamide	119	isopropyl acetate	120	methyl 3-(hydroxyl)propanoate
121	ethyl formate	122	ethyl 2-fluoroacetate	123	1-chloro-4-(methyl)benzene
124	1-(N-methylamino)ethan-1-one-2-yl	125	1-aminopropan-2-one-3-yl	126	1-aminoethan-1-one-2-yl
127	2-methylpropan-2-ol-1-yl	128	methoxymethan-yl	129	methoxyethan-yl
130	prop-1-yne-3-yl	131	methanol-yl	132	3-methylbutan-2-one-yl
133	4-methylpentan-2-one-1-yl	134	2-(methyl)-2-isopropyl-1,3-dioxolane	135	(methyl)cyclobutane
136	1,3-dimethylcyclobutane	137	1,2-dimethylcyclobutane	138	1,2,3-trimethylcyclobutane
139	2-(methyl)-2-isobutyl-1,3-dioxolane	140	2-isobutyl-1,3-dioxolane-2-yl	141	propan-2-ol-2-yl
142	hydrogen sulfide	143	Sulfur-yl	144	iodo
145	sulfur dioxide	146	hydrogen cyanidyl	147	acetonitrile
148	piperazyl	149	formamide	150	(propan-2-yl)benzene
151	(ethan-2-yl)benzene	152	furan-5-yl-2-amine	153	5-(methyl)furan-2-amine
154	furan-3-amine	155	5-methylfuran-3-amine	156	5-(methyl)thiophen-3-amine
157	thiophen-5-yl-3-amine	158	3-(ethyl)aniline	159	3-(methyl)aniline
160	4-(methyl)aniline	161	benzofuran-2-yl	162	hydrogen
163	furo[2,3-b]pyrazine	164	indazol-5-yl	165	benzo[d][1,2,3]triazol-5-yl
166	indolin-2-one-6-yl	167	N-hydroxylamine-yl	168	(hydroxyamino)methylamine
169	methanesulfonamide-yl	170	Aminosulfon-amino-yl		

**Table 6:** The calculated relative Gibbs free energies of the formation (ΔΔG_com_) of the *Mtb*TMK–TKI complex and its components were calculated for the best TKI analogs identified through the in silico screening of the virtual combinatorial library. The code names of the analogs are combined.

No.	TKI analog ^£^	∆∆H_MM_^a^	∆∆G_sol_^b^	∆∆TS ^c^	∆∆G_com_^d^	${pIC}_{50}^{pre}$ ^e^	${IC}_{50}^{pre}$ ^f^
		[kcal/mol]	[kcal/mol]	[kcal/mol]	[kcal/mol]		[nM]
Ref.	TMK1	0	0	0	0	6.02 *	950 *
1	13_13	45.41	−46.84	3.03	−4.47	6.08	836.2
2	12_13	46.34	−46.99	4.08	−4.72	6.09	821.1
3	13_5	42.46	−47.64	3.33	−8.51	6.20	625.6
4	5_58	40.31	−47.94	4.36	−11.99	6.31	487.2
5	5_149	−7.06	−45.68	7.22	−59.96	7.81	15.5
6	8_12	40.48	−43.71	5.41	−8.64	6.21	619.9
7	8_18	41.70	−47.76	3.66	−9.72	6.24	573.5
8	3_152	40.14	−45.16	6.87	−11.88	6.31	491.0
9	4_119	34.72	−44.28	6.15	−15.71	6.43	373.0
10	6_125	41.08	−44.87	7.78	−11.56	6.30	502.5
11	6_79	44.74	−45.28	7.01	−7.55	6.17	670.1
12	4_19	0.72	−44.28	2.86	−46.42	7.39	41.1
13	2_1	44.88	−48.20	5.37	−8.69	6.21	617.5
14	1_13	44.57	−47.11	2.94	−5.48	6.11	777.9
15	13_12	41.60	−47.55	3.31	−9.25	6.23	593.0
16	1_8	47.23	−48.25	−0.24	−0.78	5.96	1089.9
17	2_4	45.85	−48.49	−0.72	−1.93	6.00	1003.8
18	4_13	46.92	−47.59	6.40	−7.07	6.16	693.6
19	4_1	45.46	−47.83	0.42	−2.79	6.03	943.2
20	4_2	47.66	−48.29	1.76	−2.39	6.01	970.7
21	4_4	48.01	−48.05	5.97	−6.00	6.13	749.0
22	4_5	40.64	−47.34	4.02	−10.73	6.27	533.4
23	4_6	44.68	−47.43	5.07	−7.82	6.18	657.3
24	4_8	43.93	−47.38	1.67	−5.12	6.10	798.0
25	5_12	41.99	−49.26	2.67	−9.94	6.25	564.6
26	01_12	42.94	−47.50	−0.20	−4.35	6.07	843.2
27	1_1	46.70	−48.77	−0.38	−1.69	5.99	1021.4
28	12_12	40.74	−47.37	1.95	−8.58	6.21	622.3
29	1_2	45.86	−48.52	−1.08	−1.58	5.99	1029.6
30	10_102	35.94	−47.07	2.49	−13.63	6.36	433.2
31	10_149	35.86	−42.97	2.82	−9.92	6.25	565.1
32	10_152	38.93	−43.25	5.78	−10.10	6.25	558.1
33	11_8	42.52	−42.88	5.67	−6.03	6.13	747.7
34	14_113	24.34	−46.59	6.51	−28.75	6.84	146.1
35	14_120	28.87	−43.28	5.79	−20.21	6.57	270.0
36	8_11	45.26	−44.04	7.67	−6.45	6.14	725.3
37	8_115	46.10	−41.12	5.50	−0.52	5.95	1110.6
38	8_124	43.59	−44.09	8.74	−9.25	6.23	593.4
39	8_133	42.87	−44.34	8.32	−9.78	6.24	570.9
40	8_142	41.46	−46.68	7.82	−13.04	6.35	451.7
41	8_144	43.77	−46.31	5.35	−7.89	6.18	654.1
42	9_125	28.68	−46.00	3.38	−20.70	6.58	260.7
43	11_119	43.50	−42.35	7.81	−6.66	6.15	714.7
44	11_22	39.25	−47.35	8.53	−16.63	6.46	349.2
45	11_23	43.95	−45.34	6.60	−8.00	6.19	649.1
46	11_44	38.21	−45.05	2.33	−9.17	6.22	596.6
47	11_46	29.04	−50.98	5.98	−27.92	6.81	155.1
48	11_56	38.74	−46.66	5.78	−13.70	6.37	430.9
49	11_119	41.96	−42.18	6.28	−6.49	6.14	723.1
50	11_56	32.94	−43.65	2.02	−12.74	6.34	461.7
51	12_144	42.01	−49.06	0.64	−7.69	6.18	663.7
52	12_148	35.68	−51.18	6.23	−21.73	6.62	242.0
53	12_148	30.16	−46.47	7.55	−23.86	6.68	207.6
54	12_149	41.08	−45.96	−0.17	−4.71	6.09	822.2
55	12_154	36.10	−45.37	−0.05	−9.22	6.23	594.5
56	13_22	35.07	−50.43	8.58	−23.94	6.69	206.4
57	13_23	28.40	−40.16	8.99	−20.75	6.59	259.6
58	13_69	32.29	−44.05	4.68	−16.44	6.45	353.9
59	14_19	39.23	−46.52	11.26	−18.55	6.52	304.1
60	14_75	44.38	−46.31	8.11	−10.04	6.25	560.5
61	14_89	34.18	−44.71	3.70	−14.23	6.38	414.8
62	14_115	−10.02	−43.85	4.65	−58.53	7.76	17.2
63	15_19	24.76	−42.06	3.26	−20.56	6.58	263.2
64	15_70	20.84	−26.40	4.32	−9.88	6.25	567.0
65	16_116	39.26	−46.46	7.18	−14.39	6.39	410.2
66	16_119	35.79	−43.67	4.11	−11.99	6.31	487.1
67	16_12	40.65	−44.01	7.76	−11.12	6.29	518.7
68	16_19	47.77	−46.51	7.94	−6.68	6.15	713.4
69	16_74	47.10	−45.53	8.74	−7.17	6.16	688.8
70	16_76	34.80	−42.67	5.83	−13.70	6.37	430.9
71	16_46	−1.96	−45.49	5.25	−52.70	7.58	26.2
72	16_64	46.18	−45.21	6.26	−5.30	6.10	788.1
73	17-22	39.47	−40.93	4.14	−5.60	6.11	771.1
74	17_58	25.12	−44.27	1.86	−21.00	6.59	255.0
75	17_8	36.04	−46.88	3.78	−14.62	6.39	403.5
76	18_152	39.52	−46.06	4.45	−10.99	6.28	523.5
77	18_154	36.39	−44.08	3.76	−11.46	6.30	506.1
78	19_32	38.57	−48.25	0.04	−9.73	6.24	573.0
79	19_14	36.56	−42.71	−0.33	−5.82	6.12	759.0
80	19_101	38.04	−44.78	−2.04	−4.71	6.09	822.2
81	19_148	42.54	−46.11	3.70	−7.27	6.17	683.9
82	19_154	34.49	−43.65	−2.24	−6.92	6.15	701.2
83	19_75	38.66	−46.17	−0.39	−7.11	6.16	691.8
84	19_90	36.93	−44.94	−0.68	−7.33	6.17	681.0
85	20_87	29.68	−46.11	1.59	−18.02	6.50	315.9
86	13_1	−5.80	−59.78	1.24	−66.82	8.02	9.5
87	13_4	9.01	−72.41	2.74	−6614	8.00	9.9
88	13_6	−1.31	−60.29	3.37	−64.98	7.97	10.8
89	5_21	−2.02	−55.93	7.95	−65.89	7.99	10.1

^£^ TKI analog numbers are based on the R-groups in Table 5 for the R_1_ and R_2_ substituents (R_1__R_2_). ^a–e^ see the footnote of Table 2. ^f^ ${IC}_{50}^{pre}$ is the predicted inhibition potency toward *Mtb*TMK calculated using correlation Equation (2), Table 3; $p{IC}_{50}^{pre}$ is the negative decadic logarithm. * Experimental values of ${pIC}_{50}^{exp}$ and ${IC}_{50}^{exp}$ for the reference inhibitor TKI1 [10].

**Table 7:** Computed ADME-related properties of new TKI analogs and reference antituberculotic agents using QikProp [50].

TKI ^a^	#Stars ^b^	M_w_ ^c^ [g/mol]	S_mol_^d^ [Å²]	S_mol,hfo_^e^ [Å²]	V_mol_^f^ [Å^3^]	RotB ^g^	HB_don_ ^h^	HB_acc_^i^	logPo/w ^j^	logS_wat_^k^	logK_HSA_^l^	logB/B ^m^	BIP_Caco_^n^ [nm/s]	#metab ^o^	${IC}_{50}^{pre}$ ^p^ [nM]	HOA ^q^	%HOA ^r^
TKI1	0	477.0	785.2	236.2	1435.3	4	1	7	4.5	−6.4	0.9	−0.6	123.5	3	950 *	1	80.9
5_149	0	555.7	951.6	462.2	1762.9	4.0	1.0	9.5	4.1	−7.1	1.0	−2.6	107.4	5.0	15.5	3.0	96.3
14_115	0	612.7	957.5	524.5	1882.6	4.0	1.0	7.0	4.5	−6.3	1.0	−2.4	136.1	4.0	17.2	3.0	91.1
113_7	0	553.6	934.9	453.6	1747.5	7	2	7	5.6	−8.1	1.4	−1.7	137.3	5	146.1	3	89.1
46_11	0	620.7	955.0	490.9	1972.3	11.0	1.0	9.7	5.5	−8.2	1.3	−2.4	124.1	5.0	155.1	2.0	88.1
46_16	0	620.7	957.8	510.8	1970.7	10.0	1.0	9.7	5.5	−8.4	1.3	−2.3	128.4	4.0	26.2	3.0	89.5
13_1	0	486.5	822.0	306.3	1508.2	4.0	1.0	7.0	4.9	−4.0	1.1	−0.8	123.0	3.0	9.5	3.0	93.0
13_4	0	482.6	846.5	393.1	1549.9	5.0	1.0	7.0	5.0	−6.4	1.2	−0.9	124.2	4.0	9.9	3.0	93.5
13_6	0	498.6	844.8	394.9	1561.0	6.0	1.0	7.8	4.7	−6.5	1.0	−0.9	124.1	4.0	10.8	3.0	92.0
5_21	0	556.7	946.6	461.8	1753.8	9.0	2.0	9.0	3.1	−6.0	1.0	−2.5	130.9	4.0	10.1	2.0	80.7
Bedaquiline	4	555.5	787	213.7	1532	9	1	3.8	7.6	−7	1.7	0.4	1562.2	5		1	100
Pretomanid	0	359.3	570.6	125.4	976	5	0	5.2	2.9	−4	−0.2	−0.7	612	2		3	94.1
Lignezolide	0	337.4	586.9	357.2	1026.6	2	1	8.7	0.5	−2	−0.7	−0.6	395.2	1		3	76.3
Clofazimine	4	473.4	816.4	113	1466.6	4	1	3	8.2	−10.1	1.9	0.3	5056.8	2		1	100
Sutezolid	1	353.4	605	339.3	1060.9	2	1	7.5	1.3	−3.4	−0.4	−0.5	401.7	0		3	81.4
Delamanid	3	534.5	865.6	272.6	1536.6	7	0	6	6.1	−8.9	1.2	−1.5	284.9	2		1	80.7
Pyrazinamide	3	123.1	300.4	0	444.4	1	2	5	−0.6	−0.5	−0.8	−0.7	307.7	4		2	67.8
Gatifloxacin	0	375.4	603.7	350.6	1105.5	2	1	6.75	0.6	−4.1	0.1	−0.6	17.8	1		2	52.7
Moxifloxacin	0	401.4	614.3	374.6	1159.2	2	1	6.75	1	−4.3	0.2	−0.4	24.8	1		2	58
Rifapentine	16	828.9	1145.5	699.6	2324.6	23	4	23.5	−2.1	2	−1.2	−4.6	0	12		1	0
Ofloxacin	0	361.4	600	348.7	1060.7	1	0	7.25	−0.4	−3.2	−0.4	−0.5	23.5	1		2	49.2
Amikacin	14	614.6	822.5	341	1622	22	19	28.1	−9.4	2	−2.2	−4.8	0	15		1	0
Kanamycin	17	786.7	1179.9	431.6	2186.1	26	12	28.8	−3.8	−2.9	−2.4	−8.6	0.1	13		1	0
Imipenem	0	299.3	487	259.1	880	8	3	7.2	1	−2	−0.7	−1.4	35	3		3	61
Amoxicilline	1	365.4	639.9	170.6	1101.7	6	4.25	8	−2.4	−2.1	−1.1	−2.4	0.4	5		1	6.7
Clavulanate	2	233.2	374.4	170.4	642.2	1	1	8	−1.3	0.6	−1.4	−1	14.3	1		2	40.1

^a^ Designed TKI analogs (Table 8) and known anti tuberculosis; ^b^ drug-likeness, number of property descriptors (24 of the full list of 49 QikProp descriptors, ver. 6.5, release 139) that fall outside the range of values for 95% of known drugs; ^c^ molar mass in [g·mol^−1^] (range for 95% of drugs: 130–725 g·mol^−1^) [50]; ^d^ total solvent-accessible molecular surface, in (Å^2^) (probe radius 1.4 Å) (range for 95% of drugs: 300–1000 Å^2^); ^e^ hydrophobic portion of the solvent-accessible molecular surface, in (Å^2^) (range for 95% of drugs: 0–750 Å^2^); ^f^ total volume of the molecule enclosed by solvent-accessible molecular surface, in (Å^3^) (range for 95% of drugs: 500–2000 Å^3^); ^g^ number of rotatable non-trivial (not CX3), non-hindered (not alkene, amide, or small ring) bonds (range for 95% of drugs: 0–15); ^h^ estimated number of hydrogen bonds that would be donated by the solute to water molecules in an aqueous solution. Values are averages taken over a number of configurations, so they can assume non-integer values (range for 95% of drugs: 0.0–6.0); ^i^ estimated number of hydrogen bonds that would be accepted by the solute from water molecules in an aqueous solution (range for 95% of drugs: 2.0–20.0); ^j^ logarithm of the partition coefficient between the n-octanol and water phases (range for 95% of drugs: −2 to 6.5); ^k^ logarithm of predicted aqueous solubility, logS. S in (mol·dm^−3^) is the concentration of the solute in a saturated solution that is in equilibrium with the crystalline solid (range for 95% of drugs: −6.0 to 0.5); ^l^ logarithm of the predicted binding constant to human serum albumin (range for 95% of drugs: −1.5 to 1.5); ^m^ logarithm of the predicted brain/blood partition coefficient (range for 95% of drugs: −3.0 to 1.2); ^n^ predicted apparent Caco-2 cell membrane permeability on the Boehringer–Ingelheim scale in [nm·s^−1^] (range for 95% of drugs: <25 poor, >500 nm·s^−1^ great); ^o^ number of likely metabolic reactions (range for 95% of drugs: 1–8); ^p^ predicted inhibition constants ${IC}_{50}^{pre}$ of designed TKIs vs. *Mtb*TMK.; ^q^ human oral absorption (1 = low, 2 = medium, 3 = high); ^r^ percentage of human oral absorption in the gastrointestinal tract (<25% = poor, >80% = high); * star in any column indicates that the property descriptor value of the compound falls outside the range of values for 95% of known drugs.

**Table 8:** Ensemble averages of the total and potential energy of complexes *Mtb*TMK–TKIx for the TKI1 [10] and best new inhibitor candidates.

Compound	Chemical Structure	‹E_tot_› ^a^ [kcal/mol]	‹E_pot_› ^b^ [kcal/mol]	${IC}_{50}^{pre}$ ^c^ [nM]
TKI1	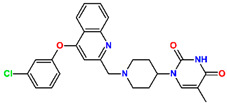	−64,403.7	−79,924.7	950 *
149_5	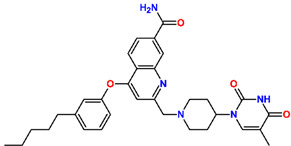	−65,314.4	−81,041.5	15.5
13_1	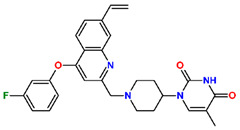	−64,371.2	−79,881.9	9.5
13_4	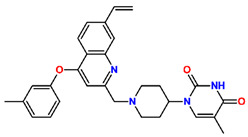	−64,405.6	−79,928.4	10
13_6	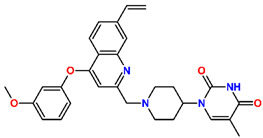	−64,538.5	−80,078.0	10.8
21_5	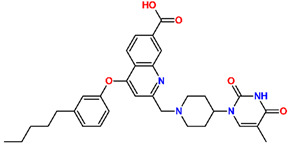	−64,641.3	−78,942.5	10.1

^a^ Ensemble average of total energy E_tot_ of the system; ^b^ ensemble average of potential energy E_pot_; ^c^ ${IC}_{50}^{pre}$ values were predicted by the QSAR model (Table 3, Equation (2)) for the new analogs. * Experimental ${IC}_{50}^{exp}$ is given for TKI1 [10]. TKI1 is the co-crystallized ligand in the crystal structure of *Mtb*TMK (PDB entry 5NRN [10,11]).

## Data Availability

The original contributions presented in this study are included in the article. Further inquiries can be directed to the corresponding author.

## Abbreviations

2D	Two-dimensional
3D	Three-dimensional
ADME	Absorption, distribution, metabolism, and excretion
ATP	Adenosine triphosphate
GFE	Gibbs free energy
∆∆G_com_	Relative GFE of formation of enzyme–inhibitor complex
∆∆G_sol_	Solvation component of the relative GFE
HBA	Hydrogen bond acceptor
HBD	Hydrogen bond donor
∆∆H_MM_	Enthalpy component of the relative GFE
HOA	Human oral absorption
HYD	Hydrophobic
HYDA	Hydrophobic aliphatic
${IC}_{50}^{exp}$	Observed half-maximal inhibitory concentration
${IC}_{50}^{pre}$	Predicted half-maximal inhibitory concentration
IE	Interaction energy
LHP	Large hydrophobic pocket
MD	Molecular dynamics
MM	Molecular mechanics
MM-PBSA	Molecular mechanics–Poisson–Boltzmann surface area
NAD	Nicotinamide adenine dinucleotide
*Mtb*	Mycobacterium tuberculosis
MtbTMK	Mycobacterium tuberculosis thymidylate kinase
PDB	Protein Data Bank
PH4	Pharmacophore model
QSAR	Quantitative structure–activity relationships
RMSD	Root mean square deviation
TB	Tuberculosis
TKIx	Inhibitors included in TS
TKIVx	Inhibitors included in VS
TS	Training set
VS	Validation set
